# Favipiravir (T-705) protects against Nipah virus infection in the hamster model

**DOI:** 10.1038/s41598-018-25780-3

**Published:** 2018-05-15

**Authors:** Brian E. Dawes, Birte Kalveram, Tetsuro Ikegami, Terry Juelich, Jennifer K. Smith, Lihong Zhang, Arnold Park, Benhur Lee, Takashi Komeno, Yousuke Furuta, Alexander N. Freiberg

**Affiliations:** 1 Department of Pathology, University of Texas Medical Branch, Galveston, USA; 2 Center for Biodefense and Emerging Infectious Diseases, University of Texas Medical Branch, Galveston, USA; 3 Sealy Center for Vaccine Development, University of Texas Medical Branch, Galveston, USA; 4 Department of Microbiology, Icahn School of Medicine at Mt. Sinai, New York, USA; 5 Toyama Chemical Co., Ltd., Toyama, Japan

## Abstract

Nipah and Hendra viruses are recently emerged bat-borne paramyxoviruses (genus *Henipavirus*) causing severe encephalitis and respiratory disease in humans with fatality rates ranging from 40–75%. Despite the severe pathogenicity of these viruses and their pandemic potential, no therapeutics or vaccines are currently approved for use in humans. Favipiravir (T-705) is a purine analogue antiviral approved for use in Japan against emerging influenza strains; and several phase 2 and 3 clinical trials are ongoing in the United States and Europe. Favipiravir has demonstrated efficacy against a broad spectrum of RNA viruses, including members of the *Paramyxoviridae*, *Filoviridae*, *Arenaviridae families*, and the *Bunyavirales* order. We now demonstrate that favipiravir has potent antiviral activity against henipaviruses. *In vitro*, favipiravir inhibited Nipah and Hendra virus replication and transcription at micromolar concentrations. In the Syrian hamster model, either twice daily oral or once daily subcutaneous administration of favipiravir for 14 days fully protected animals challenged with a lethal dose of Nipah virus. This first successful treatment of henipavirus infection *in vivo* with a small molecule drug suggests that favipiravir should be further evaluated as an antiviral treatment option for henipavirus infections.

## Introduction

Nipah virus (NiV) and Hendra virus (HeV), prototypical species of the genus *Henipavirus*, are emerging highly pathogenic paramyxoviruses which cause severe encephalitic and respiratory disease in a wide range of mammalian species, including humans^1,2^. HeV causes sporadic outbreaks in horses with human spillover in Australia, while NiV was first isolated during a large human and porcine outbreak in Malaysia and Singapore^1,3^. Almost yearly NiV outbreaks in Bangladesh and India with mortality rates averaging 70%, and a small outbreak in the Philippines have since followed^2,4–6^. Furthermore, recently discovered genetic and serologic evidence points to the presence of henipa-like viruses in African bats and spillover into humans^7,8^. The natural reservoirs for henipaviruses have been identified as fruit bats from the *Pteropus* genus^9,10^. Due to their wide host range, evidence of human-to-human spread, and the highly pathogenic nature of illness, these viruses have been proposed to have pandemic potential^11^.

Despite the pathogenicity of henipaviruses, no approved vaccines or therapeutics are available for use in humans. A subunit vaccine against HeV, which has been approved as a veterinary vaccine for use in horses in Australia, is effective in several animal models, and appears to be safe for use in humans^12–14^. Monoclonal antibodies targeting the viral envelope proteins have also shown efficacy in animal models for post-exposure prophylaxis, and have been used safely in humans under compassionate use, although their efficacy for the treatment of human disease is unknown^13,15^. The broad-spectrum antiviral ribavirin was initially used in the Malaysian outbreak in an open label trial with a reported 36% reduction in mortality^16^. However, several studies using disease-relevant animal models have repeatedly demonstrated ribavirin monotherapy as well as combination treatment with chloroquine to be ineffective at reducing the mortality of henipavirus infections^17–20^. Recently, the adenosine nucleoside analogue GS-441524, and its monophosphate prodrug GS-5734, were demonstrated to have *in vitro* antiviral activity against NiV and HeV with EC_50_ values between 0.49 to 1 μM and 0.032 to 0.055 μM, respectively^21^. Importantly, GS-5734 was protective in a non-human primate model for Ebola virus post exposure^22^ and is currently in phase 2 clinical development for the treatment of Ebola virus disease (www.clinicaltrials.gov). Additionally, another nucleoside analogue, R1479 (balapiravir), demonstrated *in vitro* antiviral efficacy against NiV and HeV with EC_50_ values of 4 μM and 2.25 μM, respectively^23^.

The viral RNA-dependent RNA polymerase (RdRp) inhibitor favipiravir (T-705; 6-flouro-3-hydroxy-2-pyrazinecarboxamine; [Avigan]) was developed by Toyama Chemical Company as an antiviral for use against influenza^24,25^. It is currently licensed in Japan for the treatment of novel or re-emerging influenza and has also undergone several phase 3 clinical trials in the United States and Europe for use against influenza (www.clinicaltrials.gov)^26^. Favipiravir acts as a purine analogue, which selectively inhibits viral RdRps^27^. In addition to its potent anti-influenza activity, favipiravir has demonstrated efficacy against a wide variety of other RNA viruses including bunyaviruses, arenaviruses, filoviruses, norovirus, flaviviruses, alphaviruses, enteroviruses, and rhabdoviruses^24,28,29^. Of note, recently completed phase 2 clinical trials for use in Ebola virus infection suggest that favipiravir treatment may result in reduced mortality when given to patients with moderate viral loads^30^. Activity against paramyxoviruses has been demonstrated *in vitro* for respiratory syncytial virus, measles virus, human metapneumovirus (hMPV), human parainfluenza virus 3, Newcastle disease virus, and avian metapneumovirus and *in vivo* against hMPV in a hamster model^25,31^. In this study, we assessed the ability of favipiravir to inhibit NiV and HeV *in vitro* as well as its efficacy in a lethal NiV-infected Syrian hamster model.

## Results

### Favipiravir inhibits henipavirus replication *in vitro*

**Figure 1:**
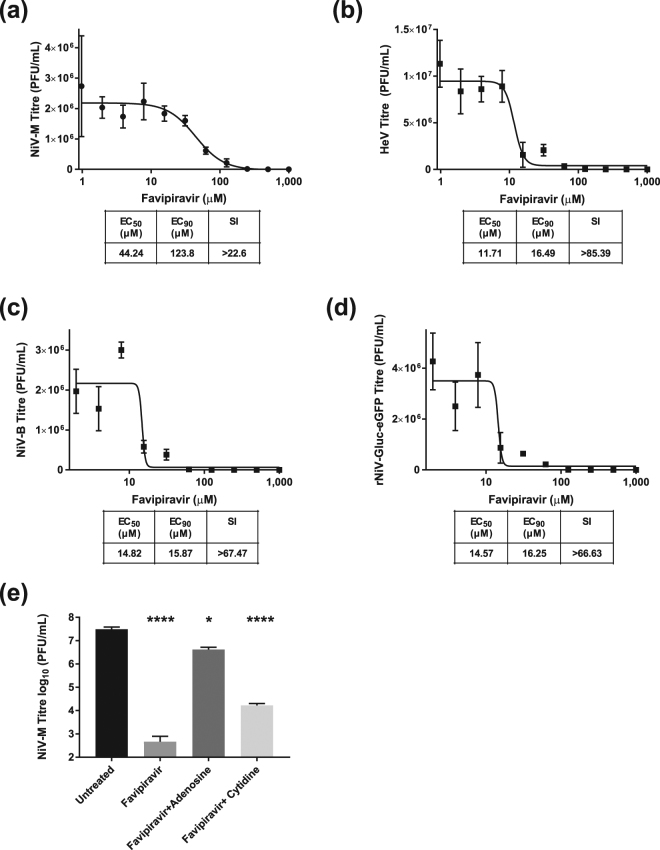
*In vitro* dose response of favipiravir against henipaviruses. Vero cells were infected with (**a**) NiV-M, (**b**) HeV, (**c**) NiV-B, (**d**) or rNiV-Gluc-eGFP at an MOI of 0.01. Cell culture media supplemented with serial 2-fold dilutions of favipiravir was added 1 hour post infection (HPI). Reduction in virus yield was determined at 48 HPI via plaque assay. (**e**) Vero cells were infected with NiV-M (MOI 0.01) and treated with 250 μM favipiravir alone or in combination with 400 μM adenosine or cytidine. Viral titres at 48HPI were then determined via plaque assay. Error bars are representing the S.D. from three individual experiments. Statistics are compared to untreated controls. *P < 0.05 and ****P < 0.0001.

To determine the inhibitory potential of favipiravir on NiV and HeV replication *in vitro*, we employed a virus yield reduction assay analysing virus titres at 48 hours post-infection (HPI). Treatment of henipavirus-infected Vero cells with favipiravir resulted in the reduction of viral titres in a dose-dependent manner for NiV-Malaysia (NiV-M), HeV, NiV-Bangladesh (NiV-B), and recombinant NiV expressing *Gaussia* luciferase and eGFP (rNiV-Gluc-eGFP) (Fig. 1a–d). Cytotoxicity was only minimal at the highest concentration tested, with a CC_50_ value of >1,000 μM (Supplemental Fig. 1). Analysis of the dose-response curves demonstrated EC_50_ values of 44.24 μM for NiV-M, 11.71 μM for HeV, 14.82 μM for NiV-B, and 14.57 μM for rNiV-Gluc-eGFP. Selective index (SI) values were >22.60 for NiV-M, >85.39 for HeV, >67.47 for NiV-B, and >66.63 for rNiV-Gluc-eGFP. EC_90_ values were 123.8 μM for NiV-M,16.49 μM for HeV, 15.87 μM for NiV-B, and 16.25 μM for rNiV-Gluc-eGFP. Additionally, we assessed if the observed inhibition was due to favipiravir’s purine analogue activity by the addition of molar excess purine or pyrimidine nucleosides (Fig. 1e). As expected, the addition of adenosine resulted in almost complete negation of favipiravir’s reduction in viral titres, while the addition of cytidine left the antiviral activity largely intact. These data demonstrate that henipaviruses are sensitive to treatment with favipiravir with EC_90_s that are consistent with those described for other paramyxoviruses and that the antiviral activity is likely due to favipiravir’s purine analogue activity^31^.

### Delayed treatment efficacy of favipiravir on Nipah virus infection *in vitro*

**Figure 2:**
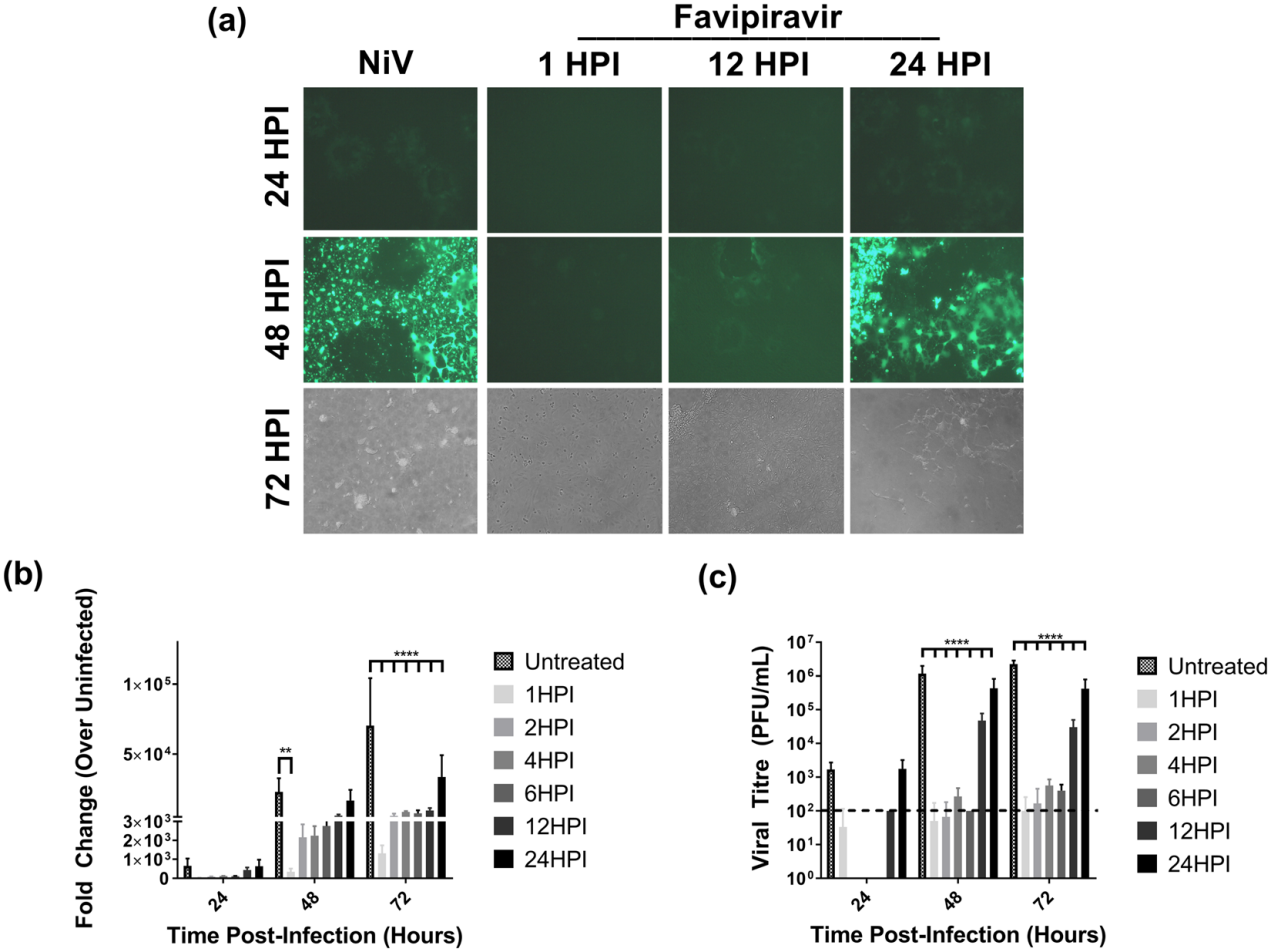
Delayed treatment *in vitro* efficacy of favipiravir against NiV infection. (**a**) Microscopic analysis of Vero cells infected with rNiV-Gluc-eGFP (MOI 0.01). Favipiravir (250 μM) was added at 1, 12, and 24 HPI; eGFP expression, development of syncytia, and cytopathic effect were monitored by microscopy at 24, 48 and 72 HPI. (**b**) and (**c**) Vero cells were infected with rNiV-Gluc-eGFP at an MOI of 0.01 and treated with 250 μM favipiravir at the indicated time points post infection. Cell culture supernatant samples were assayed for (**b**) *Gaussia* luciferase activity relative to uninfected cells and (**c**) for viral titre. Data shown in (**b**) and (**c**) represent results from two separate experiments and thus luciferase activity is normalized to uninfected cells. **P < 0.01 and ****P < 0.0001.

Next, we evaluated the inhibitory effect of favipiravir on henipavirus infection for post-exposure treatment *in vitro*. Vero cells were infected with rNiV-Gluc-eGFP, and favipiravir was added at 250 μM at 1, 12, and 24 HPI. This concentration almost completely inhibited NiV replication (>99% inhibition) in previous assays when added directly after infection at 1 HPI (Fig. 1a). Previous studies have shown rNiV expressing reporter genes such as eGFP and/or Gluc to replicate equally well when compared to *wild type* virus^8,32^. Microscopic examination of untreated infected cells revealed NiV-induced cytopathic effect (CPE) with rapid syncytia formation (Fig. 2a). By 48 HPI virtually every cell was infected and involved in syncytia formation, with almost all cells detached by 72 HPI. Addition of favipiravir at 1 HPI yielded only a few infected cells with low levels of eGFP expression, which did not further progress to syncytial formation. Cells treated at 12 HPI formed smaller syncytia compared to untreated cells, and expansion of infection and syncytia halted between 24 and 48 HPI. No GFP-positive cells were observed at 72 HPI, but small plaques were visible with light microscopy, suggesting that the syncytia had detached without further spread of infection. Cells treated with favipiravir at 24 HPI demonstrated similar CPE progression up to 48 HPI. At this time point most cells were infected, but well-defined syncytia were still visible. At 72 HPI, more cells appeared to be attached and viable compared to untreated controls.

To further refine the *in vitro* treatment window, favipiravir at 250 μM was added at six different time points between 1 and 24 HPI and every 24 hours luciferase activity was measured to determine the efficacy of favipiravir on viral gene expression (Fig. 2b). Our previous studies have confirmed a linear relationship between luciferase activity and viral titres^8^. Luciferase activity at 24 HPI revealed no significant differences between the samples. By 48 HPI the luciferase activity in the untreated controls had increased over 22,000-fold compared to uninfected cells, while cells treated with favipiravir at 1 HPI showed a significant reduction compared to untreated controls (300-fold increase relative to uninfected) at this time point, and the cells treated at 2 to 6 HPI displayed an intermediate reduction (approximately 2,000-fold increase relative to uninfected cells). By 72 HPI the luciferase activity of all treated samples was significantly lower than in the untreated controls. Addition of favipiravir to infected cells between 1 and 12 HPI resulted in reduced viral titres compared to the untreated controls (Fig. 2c). Although non-significant, titres for the treated cells at 24 HPI were below or close to the limit of detection of the plaque assay. By 48 HPI, the cells treated at 1 to 6 HPI yielded titres at or below the detection limit in contrast to untreated cells reaching titres around 10^6^ plaque forming units (PFU)/ml. Cells treated at 12 or 24 HPI also showed significant reductions in viral titres, although to a lesser degree. By 72 HPI, titres in all groups remained similar to those determined at 48 HPI. These results demonstrate that while favipiravir is most effective at inhibiting NiV replication when added immediately after infection, delaying treatment *in vitro* for up to 24 HPI still leads to a significant reduction in viral load.

### Oral administration of favipiravir fully protects from lethal Nipah virus infection in the hamster model

**Figure 3:**
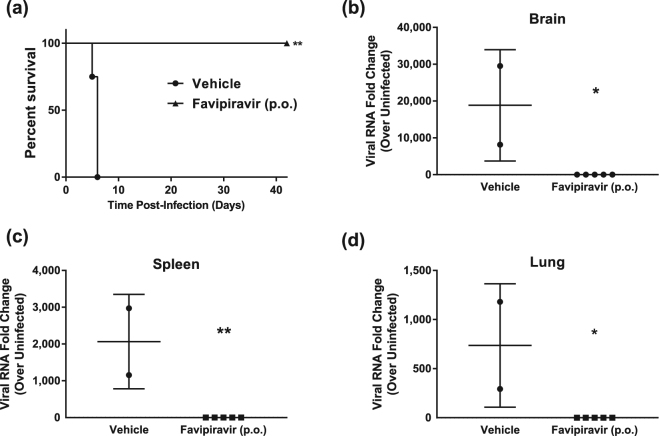
*In vivo* efficacy of orally administered favipiravir against NiV infection in Syrian hamsters. Hamsters were infected with 10^4^ PFU NiV Malaysia strain via the intraperitoneal route. Treatment with favipiravir was initiated immediately after infection. Favipiravir (*n* = 5) or vehicle (*n* = 4) was administered twice daily via oral gavage (about 12 hours apart) for 14 days. A 600 mg/kg loading dose was given on day 0, followed by 300 mg/kg/day on days 1–13 post-infection. (**a**) Survival graph of animals receiving favipiravir (black triangles) or vehicle (black circles). Graphs (**b**) to (**d**) show results from qRT-PCR analysis of tissue samples. NiV-M phosphoprotein gene copy numbers were determined in (**b**) brains, (**c**) spleens, and (**d**) lungs from euthanized control hamsters (vehicle) and survivors after favipiravir treatment. Copy numbers were quantified via comparison to a standard curve of purified NiV-M genome and normalized relative to uninfected tissues due to background detected in uninfected tissue. *P < 0.05 and **P < 0.01.

Favipiravir has been demonstrated to reduce mortality in various experimental models of viral haemorrhagic fever, encephalitic, or respiratory disease^31,33–39^. To evaluate the *in vivo* efficacy of favipiravir against NiV-M, we utilized the Syrian hamster model which closely mirrors most aspects of human disease, such as widespread vasculitis, pneumonia, and encephalitis and has been widely accepted for the evaluation of antiviral therapeutics and vaccine candidates^19,20,40–45^. Hamsters were infected with a lethal dose of 10^4^ PFU NiV-M via the intraperitoneal (i.p.) route similar to previous studies and treatment was initiated immediately after infection^19,46^. Favipiravir was administered twice daily via the perioral (p.o.) route for 14 days, again similar to previous studies evaluating the antiviral activity of favipiravir^37,38,47^. On challenge day, a loading dose of 600 mg/kg/d was administered immediately after infection, followed by 300 mg/kg/d on days 1–13. Control animals were dosed according to the same schedule with vehicle only. All vehicle-treated NiV-infected animals uniformly developed clinical signs of disease including hyperreflexia, ataxia, irregular breathing, and lethargy and succumbed to disease or were humanely euthanized on days 5 or 6 PI (Fig. 3a). Animals treated with favipiravir did not develop clinical signs of disease during the course of the study through 42 days post infection (DPI; Fig. 3a). Furthermore, weight data revealed steep weight loss prior to death or euthanasia in vehicle-treated animals, while favipiravir-treated animals steadily gained weight throughout the duration of the study (Supplemental Fig. 2a). Virus titrations from tissues were inconclusive as we were only able to recover viable virus in one of the four vehicle-treated animals, but no virus was detected in the favipiravir-treated group (data not shown). Real time RT-PCR for the viral P gene was conducted on brains, spleens, and lungs to compare viral load between moribund animals that were euthanized and survivors. As expected, high levels of viral P gene expression were detected in all three tissues in the vehicle-only controls compared to favipiravir-treated animals, where no viral RNA was detected (Fig. 3b–d). Two of five survivors developed neutralizing antibody titres (PRNT_50_s) of >80 and >1280, respectively, while the remaining three survivors had titres of <20 (Supplemental Table 1). These results demonstrate that favipiravir administered twice daily p.o. beginning immediately after infection is highly efficacious in preventing NiV-induced morbidity and mortality in the hamster model.

### Administration of favipiravir subcutaneously protects hamsters from lethal Nipah virus infection

**Figure 4:**
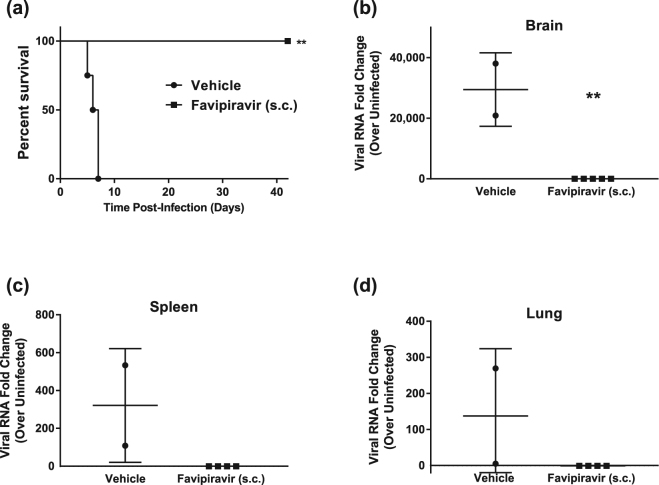
*In vivo* efficacy of subcutaneously administered favipiravir against NiV infection in Syrian hamsters. Hamsters were infected with 10^4^ PFU NiV Malaysia strain via the intraperitoneal route. Treatment with favipiravir was initiated immediately after infection. Favipiravir (*n* = *5)* or vehicle (*n* = 4) was administered once daily via the subcutaneous route for 14 days. A 600 mg/kg loading dose was given on day 0, followed by 300 mg/kg/day on days 1–13 post-infection. (**a**) Survival graph of animals receiving favipiravir (black squares) or vehicle (black circles). Graphs (**b**) to (**d**) show results from qRT-PCR analysis of tissue samples. NiV-M phosphoprotein gene copy numbers were determined in (**b**) brains, (**c**) spleens, and (**d**) lungs from euthanized control hamsters (vehicle) and survivors after favipiravir treatment. Copy numbers were quantified via comparison to a standard curve of purified NiV-M genome and normalized relative to uninfected tissues due to background detected in uninfected tissue. **P < 0.01.

To determine the efficacy of once daily subcutaneous (s.c.) administration of favipiravir, such as recently used in a Lassa virus (LASV) guinea pig model^33^, hamsters were infected with a lethal dose of 10^4^ PFU NiV-M via the i.p. route and treatment was initiated immediately after infection. Similar to the oral administration study described above, a loading dose of 600 mg/kg/d was administered immediately after infection, followed by 300 mg/kg/d on days 1–13. All vehicle-treated animals became ill within 7 DPI and displayed signs of paralysis, ataxia, and irregular breathing (Fig. 4a). Favipiravir-treated animals survived until the end of the study (42 DPI) with no development of clinical signs of disease and steadily gained weight throughout the course of the experiment (Supplemental Fig. 2b). As with the previous study, attempts at virus titration from tissue were inconclusive, and high loads of the viral P gene were detected by RT-PCR in all three tissues examined in non-treated animals (Fig. 4b–d), while the viral load in all survivors was not detectable. Of the five surviving animals, three developed neutralizing antibody titres (two > 80 and one > 20), while the remaining two survivors had titres of <20 (Supplemental Table 1). These results demonstrate that administration of favipiravir s.c. once daily beginning immediately after infection is also highly efficacious in preventing NiV-induced morbidity and mortality in the hamster model.

### Favipiravir treatment results in decreased viral antigen and histopathological changes

**Figure 5:**
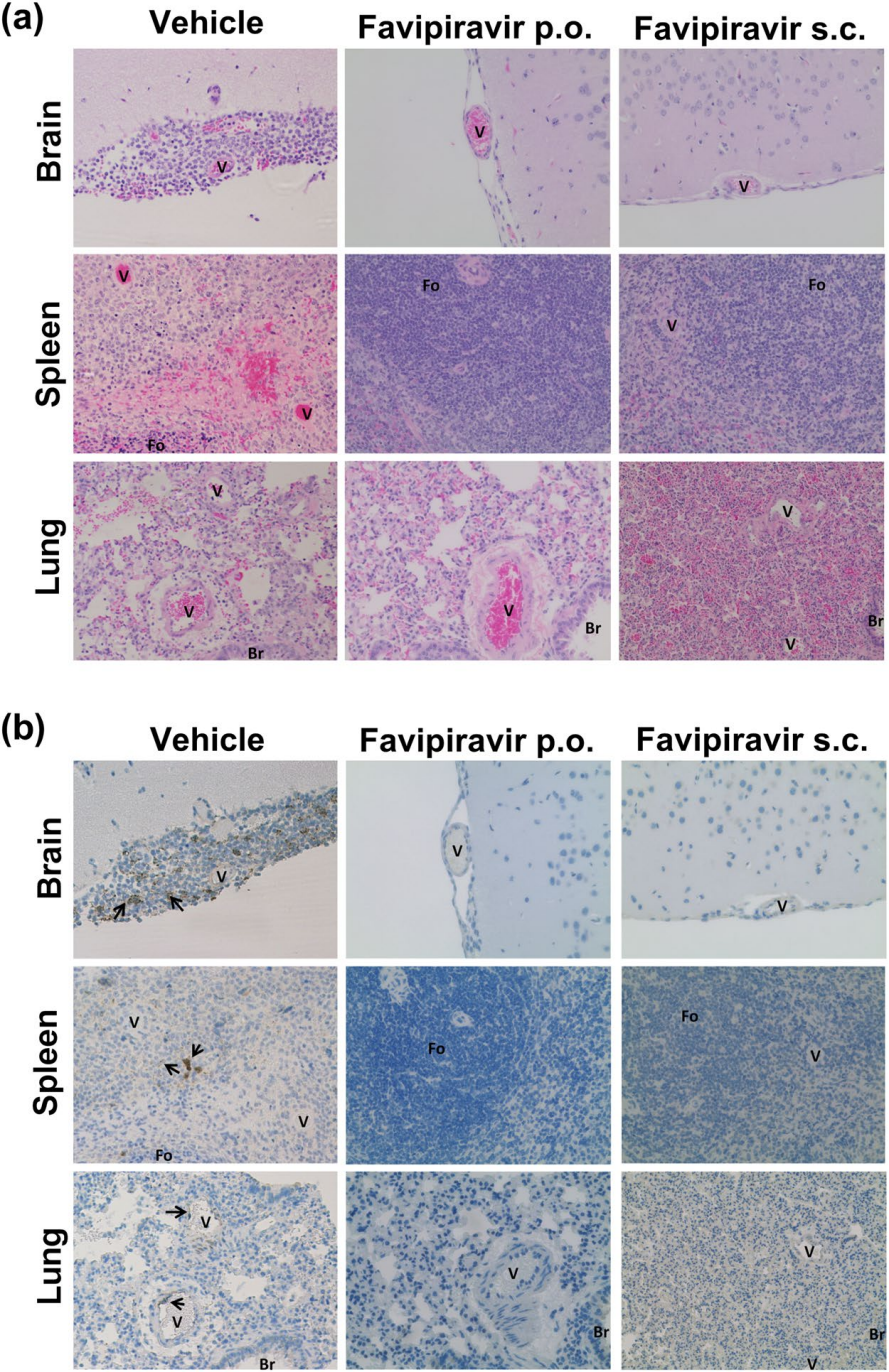
Histopathology and immunohistochemistry. (**a**) Formalin fixed tissues were embedded in paraffin and H&E stained. Images represent brains, lungs, and spleens of vehicle control animals, favipiravir p.o. and favipiravir s.c. (**b**) Formalin fixed tissues were embedded in paraffin and stained with anti-NiV nucleoprotein antibodies. Images represent brains, lungs, and spleens of vehicle control animals, favipiravir p.o. and favipiravir s.c. V, blood vessels; Br, bronchioles; Fo, follicles; Arrows, NiV nucleoprotein antigens.

In order to determine the pathological changes present in favipiravir-treated NiV-M-infected hamsters we examined brain, spleen, and lung collected from euthanized animals during the study and survivors at 42 DPI via H&E stains (Fig. 5a) and IHC (Fig. 5b) against NiV nucleoprotein. Vehicle-treated animals displayed characteristic pathological lesions of NiV infection (Fig. 5)^13,15^: Lungs displayed perivascular infiltration of inflammatory cells, and NiV antigens were detected in endothelial cells, which occasionally formed syncytia, as well as in smooth muscle cells of pulmonary vessels. Mild to moderate interstitial pneumonia with alveolar edema or haemorrhage and occasional increase in type II pneumocytes were also seen. In the spleen, follicles were less visible, and the red pulp cord displayed necrotic areas scattered with mononuclear or reticular cells with NiV antigens. In brains, meningitis with an infiltration of neutrophils and mononuclear cells was found, and viral antigens were detected in mononuclear cells with elongated cytoplasm in meninges and occasionally in neurons in parenchyma. Tissues of animals which were treated with favipiravir, either p.o. or s.c., were similar: no prominent findings of diseases were detected in brains, lungs, and spleens in the H&E sections (Fig. 5a). None of the treated hamsters displayed detectable NiV antigens in brains, lungs, or spleen spleens (Fig. 5b). Lungs of treated hamsters did not show cellular infiltration in pulmonary blood vessels, although mild consolidation of lung parenchyma was observed (Fig. 5a).

## Discussion

Despite the high virulence of henipaviruses, no therapeutics are approved for human use. Several approaches have shown efficacy in animal models: monoclonal antibodies against the envelope F and G proteins appear to be effective post-exposure treatments in various animal models^15,42,48,49^, and peptides derived from the C-terminal heptad repeat of the F protein inhibit fusion and viral entry *in vitro* and reduce mortality *in vivo*^45,50^. However, none of these have been moved forward into human clinical trials. Furthermore, the unpredictable and rare nature of human henipavirus infection requires quick deployment of antiviral compounds for use in rural areas in developing countries, where monoclonal antibody and peptide therapies may not be feasible. A small molecule antiviral, on the other hand, could be more easily stockpiled and would not rely on a cold-chain for rapid deployment. In Malaysia, ribavirin was evaluated in an open label trial reporting a 36% reduction in mortality^16^. However, this study used historical controls, was small in nature, and was not blinded or randomized, clouding the true efficacy. Several *in vivo* studies have failed to demonstrate efficacy of ribavirin and or chloroquine against henipavirus infection across various animal models^17–20^. We hypothesized that favipiravir, due to its ability to inhibit a wide range of RNA viruses – including several highly pathogenic RNA viruses – and demonstrated safety in humans, may be an effective therapy against henipaviruses, which could be applied during outbreaks.

In the present proof-of-concept study we demonstrate data establishing *in vitro* and *in vivo* efficacy of favipiravir against henipaviruses. *In vitro* studies showed similar efficacies to those seen in other paramyxoviruses^31^. Additionally, *in vivo* studies using the well-characterized Syrian hamster model of NiV-M infection demonstrate for the first time the ability of a small molecule, favipiravir, to prevent NiV-induced morbidity and mortality when administered directly after infection. These promising results warrant further evaluation of favipiravir for the treatment of henipavirus infection.

*In vitro*, favipiravir displayed EC_50_s of 44.24 μM, 11.71 μM, 14.82 μM, and 14.57 μM and EC_90_s of 123.8 μM, 16.49 μM, 15.87 μM, and 16.25 μM against NiV-M, HeV, NiV-B, and rNiV-Gluc-eGFP respectively (Fig. 1). The EC_90_ against HeV and NiV-B is comparable to those recently determined for other paramyxoviruses (2–55 μM), while the EC_90_ against NiV-M is higher^31^. The L genes of the two viruses share only 87% amino acid homology, suggesting that potential structural differences in the polymerase may make HeV more susceptible to favipiravir. Interestingly, the EC_90_ of rNiV-Gluc-eGFP is lower than NiV-M despite the backbone of this strain being derived from NiV-M. It is possible that the addition of reporter genes slightly attenuates the recombinant strain with regards to its susceptibility to antivirals, although the difference remains small. Additionally, we affirmed via nucleoside supplementation assays that favipiravir’s mechanism of action is likely as a purine analogue.

In time of addition experiments *in vitro* we demonstrated that delay of treatment for up to 6 hours after NiV infection resulted in essentially complete inhibition of viral replication as measured by viral titre and viral transcription of the reporter gene luciferase. Treatment delays of 12 and 24 hours still resulted in significantly reduced luciferase activity and virus titres compared to untreated controls (Fig. 2b,c). Viral titres and luciferase activity were closely correlated throughout the experiment.

Previous *in vivo* studies have established a standard dosing route and dose for hamsters of 300 mg/kg/d favipiravir p.o.^51,52^. In the current study, dosing NiV-M-infected hamsters with 300 mg/kg/d twice daily p.o. resulted in complete survival and no obvious signs of morbidity, such as weight loss, clinical disease or behavioural changes (Fig. 3a). While virus titrations were inconclusive, qRT-PCR demonstrated the lack of detectable viral RNA in all tissues tested in favipiravir-treated animals compared to untreated controls (Fig. 3b–d).

Favipiravir has been designed as a drug for oral delivery^24^, and twice daily oral administration most closely resembles the treatment regiments currently approved for human use. However, under biosafety level (BSL)-4 conditions animals must be anesthetized for every dosing, and twice daily dosing may thus place added stress on infected animals. Therefore, we also evaluated a once daily s.c. dosing regimen similar to one which has recently been shown to be highly efficacious in a lethal LASV guinea pig model^33^. As in our oral dosing study, hamsters that were dosed once daily with 300 mg/kg/d of favipiravir s.c. displayed 100% survival and no obvious morbidity after lethal NiV-M challenge (Fig. 4a). Similarly, all tissues in favipiravir-treated animals appeared negative for viral RNA (Fig. 4b–d).

Either favipiravir treatment regimen greatly reduced histopathological changes in brains, lungs, and spleens (Fig. 5a). Moreover, viral antigens were not detectable in those treated animals (Fig. 5b). The only changes noted were mild consolidation in the lungs. Previous studies have not looked at the long term pathological outcomes of favipiravir treatment of acute viral diseases, although several have shown that acute pathological injuries can still occur during viral infection with favipiravir treatment^33,34,37,53^. While both NiV and HeV have been shown to persist in the CNS and to cause neurologic relapse in a low percentage of recovered patients^54,55^ we observed no changes in brain histology and noted the absence of viral antigen in brains of treated animals.

Together, the results presented in this study clearly demonstrate the *in vitro* and *in vivo* efficacy of favipiravir against highly pathogenic henipaviruses, and provide a foundation for further studies regarding the optimization of doses, routes, and timing of treatment after infection. Additionally, while *in vivo* efficacy was demonstrated for NiV-M, efficacy must be confirmed against HeV and NiV-B. Further studies are also required to assess the post-exposure efficacy of favipiravir when added at delayed treatment times after infection. Our data indicate that favipiravir is a small molecule antiviral candidate for potential post-exposure prophylaxis for lab workers, healthcare providers, or patient contacts potentially exposed to Nipah virus.

## Methods

### Cells and viruses

Vero CCL81 cells were purchased from the American Type Culture Collection (ATCC, Manassas, VA). Cells were grown and maintained in minimal essential media (MEM) supplemented with 10% fetal bovine serum (FBS) at 37 °C under 5% CO_2_. FBS was lowered to 2% during viral infections.

NiV (Malaysia and Bangladesh (NiV-M and NiV-B, respectively) strain) was provided by the Special Pathogens Branch (Centers for Disease Control and Prevention, Atlanta, GA, USA) and HeV (prototype strain) was provided by the Special Pathogens Program (National Microbiology Laboratory, Canadian Science Centre for Human and Animal Health, Winnipeg, Canada). rNiV-Gaussia luciferase and green fluorescent protein (rNiV-Rbz-NP-Gluc-p2A-eGFP; here abbreviated as rNiV-Gluc-eGFP) was rescued as described previously^32^. Virus titres were determined by plaque assay on Vero cells and indicated as PFU/ml. All work with infectious virus was conducted in the Robert E. Shope BSL-4 or Galveston National Laboratory BSL-4 laboratories at the University of Texas Medical Branch (UTMB).

### Compounds

Favipiravir was provided by Toyama Chemical Company, Ltd. (Toyama, Japan). For *in vitro* studies, favipiravir was dissolved in MEM. For *in vivo* studies, favipiravir was dissolved in 0.5% carboxymethylcellulose (Sigma Aldrich) for dosing via oral gavage (p.o.) and in 74.6 mg/ml meglumine solution (Sigma Aldrich) at pH 8.5 for dosing via the subcutaneous (s.c.) route.

### *In vitro* virus yield reduction assay

Vero cells were infected with each virus at a multiplicity of infection (MOI) of 0.01 for one hour. Virus was removed; cells washed with DPBS, and overlaid with fresh MEM supplemented with 2% FBS and 2-fold dilutions of favipiravir (1,000 to 0.98 μM). Supernatant samples were collected at 48 hours post-infection (HPI) and titrated. Virus yield reduction was calculated as percent reduction of viral titres compared to untreated controls. Cellular cytotoxicity of favipiravir was determined in the absence of viral infection using a neutral red based *in vitro* toxicology assay kit (Sigma-Aldrich) The 50% effective concentration (EC_50_) and 50% cell cytotoxic dose (CC_50_) were determined as the favipiravir concentration at which viral titres were 50% of the untreated controls at the respective time point and the favipiravir concentration leading to 50% cytotoxicity and were calculated using regression analysis. The selectivity index (SI) was calculated using the formula SI = CC_50_/EC_50_.

### *In vitro* time of addition experiments

Vero cells were infected with rNiV-Gluc-eGFP (MOI of 0.01). At one HPI, inoculum was removed, cells washed with DPBS, and fresh media added. Favipiravir was added at 250 µM at 1, 2, 4, 6, 12, and 24 HPI and supernatant was sampled at 24, 48, and 72 HPI. Cells were imaged by fluorescence microscopy at the indicated time points. Viral titres were determined via plaque assay, and *Gaussia* luciferase activity was measured using the Biolux® Gaussia luciferase assay kit (New England Biolabs, Ipswich, MA) following the manufacturer’s protocol. Luciferase readings were recorded using a Modulus™ luminometer (Turner Biosystems, Sunnyvale, CA). All experiments were performed in biological triplicates and figures are reported in fold change to compare two separate experiments.

### Nucleoside supplementation assay

Vero cells were infected with NiV-M (MOI of 0.01). At 1 HPI, cells were treated with either mock treatment, 250 μM favipiravir, 250 μM favipiravir with 400 μM adenosine (Sigma-Aldrich), or 250 μM favipiravir with 400 μM cytidine (Sigma-Aldrich). At 48 HPI supernatant was collected and titrated via plaque assay.

### Animals and ethics statements

Female Syrian hamsters (4–5 weeks old, 70–100 g) were purchased from Harlan Laboratories. All procedures were conducted under animal protocols approved by the UTMB Institutional Animal Care and Use Committee and complied with USDA guidelines in an AAALAC-accredited lab. Animals were housed in microisolator caging equipped with HEPA filters in the BSL-4 laboratories.

### Hamster efficacy studies

Hamsters were challenged with 10^4^ PFU of NiV-M by intraperitoneal (i.p.) injection. Animals (n = 5) received 300 mg/kg/d favipiravir p.o. every 12 hrs, initiated immediately after infection and continued twice daily until 13 days post-infection (DPI). Animals received a loading dose of 600 mg/kg/d on the challenge day, and a 300 mg/kg/d maintenance dose thereafter.

In a second experiment, animals (n = 5) received 300 mg/kg/d favipiravir once daily s.c. Dosing was initiated immediately after infection with a 600 mg/kg/d loading dose and a 300 mg/kg/d maintenance dose thereafter until 13 DPI. In each experiment, a virus-only control group (n = 4) received the relevant vehicle solution only.

Animals determined to be moribund via daily clinical scoring or displaying greater than 20% weight loss were euthanized. Blood was collected via terminal cardiac puncture, and tissues were formalin fixed for immunohistochemical (IHC) studies or homogenized in PBS for viral titre measurements, or homogenized in TRIzol reagent (Life Technologies, Carlsbad, CA) for qRT-PCR. Survivors were monitored up to day 42 at which point they were euthanized and serum and tissues were collected. For each manipulation (viral infection or drug administration), animals were anesthetized with isoflurane (Piramal, Bethlehem, PA).

### Histopathology

Formalin fixed tissues were embedded in paraffin at the UTMB Research Histopathology Core. Embedded tissues were sectioned and stained with haematoxylin and eosin (H&E). Additional sections underwent IHC staining with rabbit anti-NiV N antibody (Dr. Basler, Georgia State University) at a dilution of (1:1,000), an HRP-conjugated anti-rabbit antibody and counterstained with haematoxylin. Images were obtained using an Evos XL Core microscope (Life Technologies).

### qRT-PCR

qRT-PCR was used to quantitate viral loads in tissues. RNA was extracted from tissues homogenized in TRIzol reagent using Direct-zol RNA Miniprep kits (Zymo Research, Irvine, CA). qRT-PCR assays were then run using QuantiFast RT-PCR mix (Qiagen, Hilden, Germany), probes targeting NiV-M P gene (5′-ACATACAACTGGACCCARTGGTT-3′ and 5′-CACCCTCTCTCAGGGCTTGA-3′) (IDT, Coralville, IA), and fluorescent probe (5′-6FAM-ACAGACGTTGTATA + C + CAT + G-TMR) (TIB MOLBIOL, Adelphia, NJ). qRT-PCR was performed using the following cycle: 10 minutes at 50 °C, 5 minutes at 95 °C, and 40 cycles of 10 seconds at 95 °C and 30 seconds at 60 °C using a BioRad CFX96 real time system. Assays were run in parallel with uninfected hamster tissues and a NiV-M stock standard curve. Gene copies were quantified based on a standard curve and fold changes compared to uninfected tissue were reported due to background detected in uninfected tissues.

### Neutralizing antibody titres

Neutralizing antibodies from moribund animals or survivors were measured using a plaque reduction neutralization test (PRNT). Serum was gamma-irradiated (5 Mrad), followed by heat inactivation at 56 °C for 30 minutes, then diluted in series in MEM with 2%FBS, and incubated with a dose of 50 PFU NiV-M for one hour. Vero cells were infected with the virus:serum mix and a standard plaque assay was conducted. PRNT_50_ was defined as the antibody dilution yielding 50% reduction in plaques (±10%) and PRNT_90_ was defined as the titre at which there was a 90% (±10%) reduction in plaques.

### Statistical analysis

All statistical analysis was completed using Prism (GraphPad Software, La Jolla, CA). Comparisons of viral titres and luminescence were subjected to a two-way repeated measure analysis of variance (ANOVA) with a Tukey post-test. Dose response curves were developed using nonlinear regression. Survival curves were compared using the Mantel-Cox log-rank test. Fold changes in viral gene copies were compared using an unpaired t-test.

### Data availability statement

The datasets generated during the currently study are available from the corresponding author on reasonable request.

## Electronic supplementary material

Supplementary Information
