# Supplementary material for: Favipiravir (T-705) protects against Nipah virus infection in the hamster model
Source: Sci Rep. 2018 May 15;8:7604. doi: 10.1038/s41598-018-25780-3 (PMC5954062; doi:10.1038/s41598-018-25780-3)
Supplement: Supplementary file 1 — Supplementary Information [file 41598_2018_25780_MOESM1_ESM.pdf]

Favipiravir (T-705) protects against Nipah virus infection in the hamster model

Brian E. Dawes[1], Birte Kalveram[1], Tetsuro Ikegami[1,2,3], Terry Juelich[1], Jennifer K. Smith[1], Lihong Zhang[1], Arnold Park[4], Benhur Lee[4], Takashi Komeno[5], Yousuke Furuta[5], Alexander N. Freiberg[1,2,3,*]

[1]Department of Pathology, University of Texas Medical Branch, Galveston, USA

[2]Center for Biodefense and Emerging Infectious Diseases, University of Texas Medical Branch, Galveston, USA

[3]Sealy Center for Vaccine Development, University of Texas Medical Branch, Galveston, USA

[4]Department of Microbiology, Icahn School of Medicine at Mt. Sinai, New York, USA

[5]Toyama Chemical Co., Ltd., Toyama, Japan

*Corresponding author: Alexander N. Freiberg, Department of Pathology, University of Texas Medical Branch, 301 University Boulevard, Galveston, 77555-0609, Tel.: +1-409-772-2882, anfreibe@utmb.edu

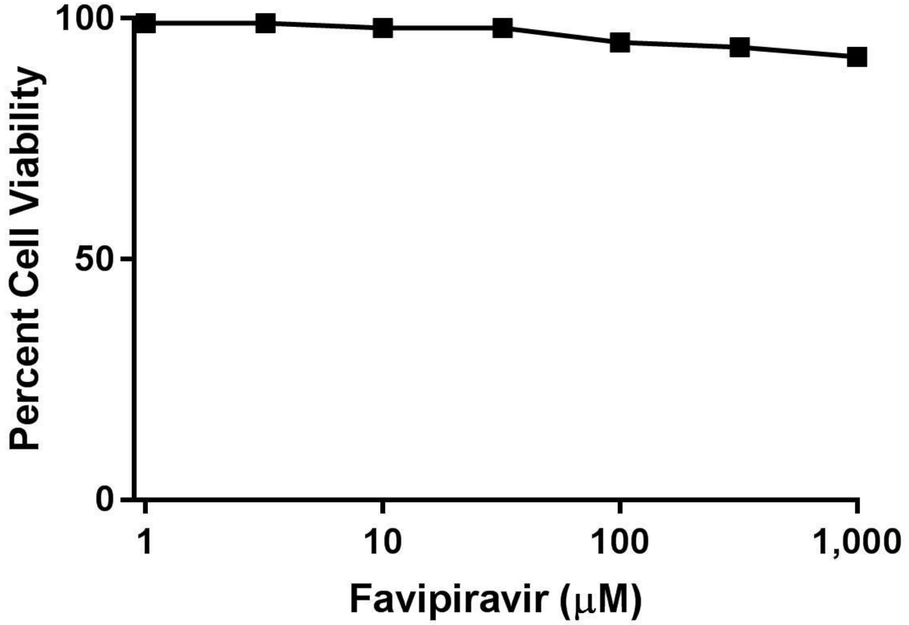

Supplemental Figure 1. Vero cells were treated with two-fold dilutions of favipiravir and cell viability was assessed using a neutral red assay. CC_{50} was determined to be >1,000 μM.

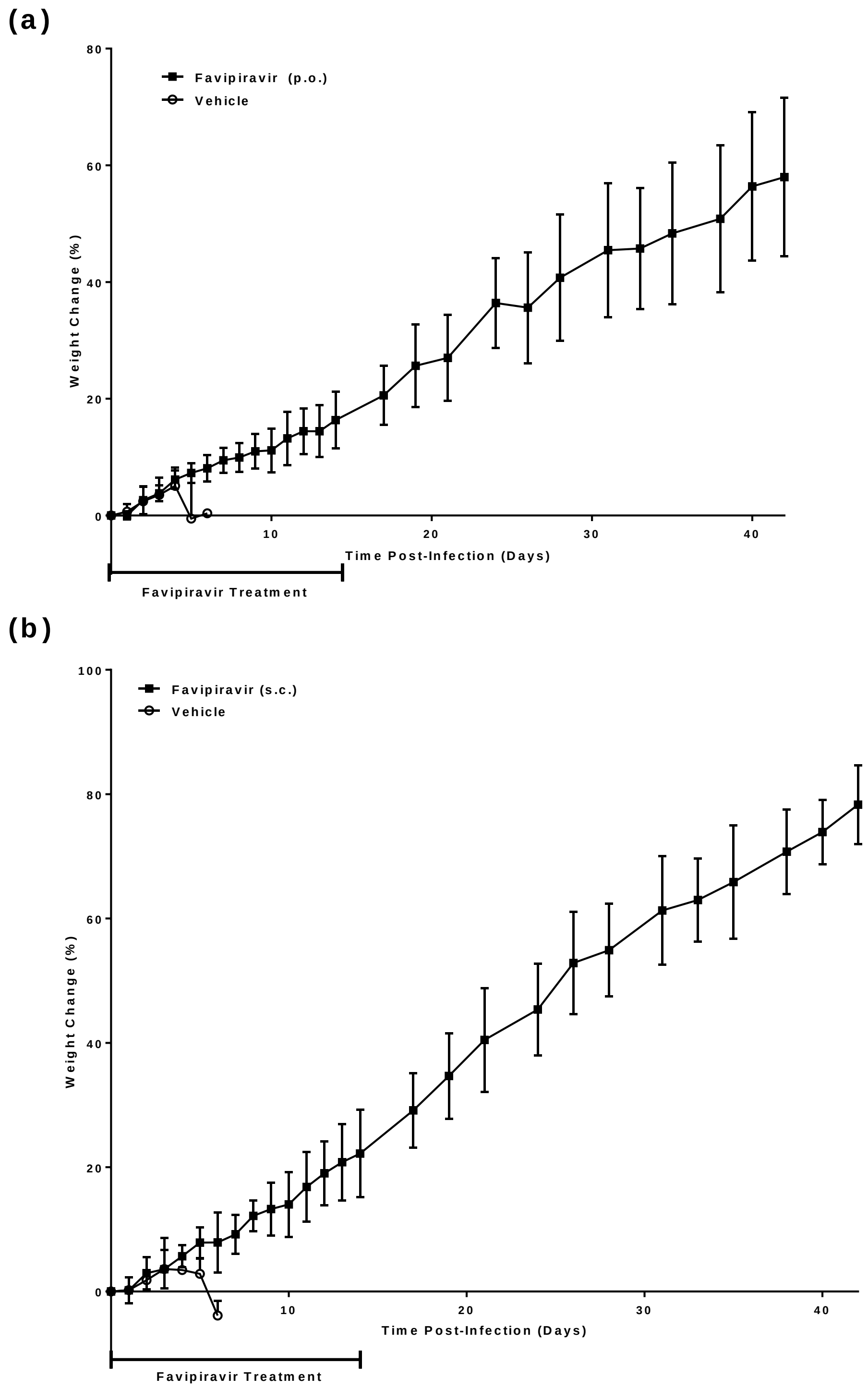

Supplemental Figure 2. Weights of infected hamsters undergoing **(a)** p.o. or **(b)** s.c. treatments. Weights were monitored daily for all infected animals through day 14 and every other day thereafter.

Group	$PRNT_{50}$	$PRNT_{90}$
**Favipiravir p.o.**	**<20**	**<20**
	**80**	**20**
	**<20**	**<20**
	**1280**	**320**
	**<20**	**<20**
**Favipiravir s.c.**	**<20**	**<20**
	**80**	**20**
	**<20**	**<20**
	**80**	**20**
	**20**	**<20**

Supplemental Table 1. Neutralizing antibody titers from *in vivo* efficacy studies. Serum collected from survivors was gamma irradiated and heat inactivated. Serum was then diluted and incubated with 50PFU NiV-M prior to infection of Vero CCL81 cells. Cells were incubated for 3 days and plaques were quantified for calculations of $PRNT_{50}$ and $PRNT_{90}$.
